# Changes in Water Properties in Human Tissue after Double Filtration Plasmapheresis—A Case Study

**DOI:** 10.3390/molecules27123947

**Published:** 2022-06-20

**Authors:** Felix Scholkmann, Roumiana Tsenkova

**Affiliations:** 1 Biomedical Optics Research Laboratory, Department of Neonatology, University Hospital Zurich, University of Zurich, 8091 Zurich, Switzerland; 2 Aquaphotomics Research Department, Graduate School of Agricultural Science, Kobe University, Kobe 657-8501, Japan; rtsen@kobe-u.ac.jp

**Keywords:** double-filtration plasmapheresis, INUSpheresis, near-infrared spectroscopy, aquaphotomics, water

## Abstract

Double-filtration plasmapheresis (DFPP) is a blood cleaning technique that enables the removal of unwanted substances from the blood. In our case study, we performed near-infrared (NIR) spectroscopy measurements on the human hand tissue before and after a specific DFPP treatment (INUSpheresis with a TKM58 filter), along with NIR measurements of the substances extracted via DFPP (eluate). The spectral data were analyzed using the aquaphotomics approach. The analysis showed that the water properties in the tissue change after DFPP treatment, i.e., an increase in small water clusters, free water molecules and a decrease in hydroxylated water as well as superoxide in hydration shells was noted. The opposite effect was observed in the eluates of both DFPP treatments. Our study is the first that documents changes in water spectral properties after DFPP treatments in human tissue. The changes in tissue water demonstrated by our case study suggest that the positive physiological effects of DFPP in general, and of INUSpheresis with the TKM58 filter in particular, may be associated with improvements in water quality in blood and tissues.

## 1. Introduction

Introduced in the early 1980s by Agishi et al. [1], double-filtration plasmapheresis (DFPP) allows the removal particles in blood plasma having sizes between the pore size of the first (plasma separator) and second filter membrane (plasma fractionator). This is realized by first separating the blood into plasma and blood cells (with the plasma separator) and then fractioning the separated plasma into large and small molecular weight components. Although the blood cells and plasma with small molecular weight components are transfused back to the patient, the large molecular weight components (eluate) are filtered out. DFPP is an effective, efficient and patient-friendly blood purification procedure.

The DFPP technique allows pathophysiological relevant molecules (e.g., circulating autoantigens, autoantibodies, circulating immune complexes, damaged proteins) and toxins (e.g., environmental toxins and toxins from microorganisms) to be removed from the blood of a subject. This blood cleaning procedure has been successfully used therapeutically in many diseases [2,3,4], including myasthenia gravis [5,6,7,8,9,10,11,12], chronic inflammatory demyelinating polyneuropathy [12], anti-glomerular basement membrane disease [13], hypoglycemia and hyperglycemia induced by insulin antibodies [14], pancreatitis induced by hypertriglyceridemia [15,16,17], Guillain–Barré syndrome [12,18,19,20,21], Crow–Fukase syndrome [12], rheumatoid arthritis [22,23,24], chronic hepatitis C [25,26], pemphigus [27,28], bullous pemphigoid [29,30], atopic dermatitis [31], dermatomyositis [12], polymyositis [12], membranous nephropathy [32], acute thallotoxicosis [33], antibody-associated vasculitis [34,35,36], antisynthetase syndrome [37], diffuse proliferative lupus nephritis [38], refractory chronic urticaria [39], systemic lupus erythematosus associated with autoimmune thyroid disease [40], rhesus D-incompatible pregnancy [41], anti-PP1Pk isoantibodies-incompatible pregnancy [42], adult onset Still’s disease [43], multiple sclerosis [12,44,45,46], Eaton–Lambert syndrome [12], hemorrhagic fever [47], acquired thrombotic thrombocytopenic purpura [48], neuromyelitis optica [49,50], Graves’ disease [51], antiphospholipid syndrome [52], age-related macular degeneration [53], diffuse cutaneous systemic sclerosis [54], co-infection infection with Hepatitis C and human immunodeficiency virus [55], acute atherothrombotic brain infarction [56], cryoglobulinemia [57], inflammatory polyneuropathy [58], chronic inflammatory demyelinating polyradiculoneuropathy [59], prevention of antibody-dependent xenograft rejection [60,61] and even cancer [62].

Although DFPP has been widely and routinely used in clinical practice in Asia, especially in Japan, for decades, its use is not yet widespread in the West. For a few years now, however, DFPP has been attracting increasing attention in Europe, largely due to the development of a specific type of DFPP, called INUSpheresis, by developers and scientists from Germany. For example, these researchers have recently shown that this type of DFPP has great therapeutic potential for the treatment of metabolic and non-metabolic peripheral neuropathy [63], borreliosis [64], Alzheimer’s disease [65] and chronic post-COVID-19 syndrome (“long-COVID”) [66].

In the case of the treatment of neuropathy patients [63], a significant reduction in total cholesterol, triglycerides, LDL-cholesterol, serum C-reactive protein (sCRP), tumor necrosis factor-α (TNF-α), eosinophilic cationic protein (ECP), fibrinogen and the chemokine RANTES (Regulated And Normal T cell Expressed and Secreted) could be achieved. Furthermore, a significant amount of environmental toxins (including heavy metals and pesticides) in the blood of neuropathy patients could be removed by applying this type of DFPP. Such an improvement in the lipid profile and in inflammatory markers was also evident in borreliosis patients treated with this type of DFPP [64], including a decrease in the inflammatory lipid lipoprotein-associated phospholipase A2 (Lp-PLA2). A clinical improvement in the patients was also evident. In case of Alzheimer’s disease patients treated with this type of DFPP [65], a significant reduction in the concentration of RANTES, fibrinogen, sCRP, ECP, TNF-α, and α2-macroglobulin (a marker of neuronal injury and generally increased in Alzheimer’s disease [67,68]) was evident. A significant amount of toxins (e.g., aluminum and organophosphorus pesticides) could be also removed from the blood of these patient with this procedure. In another recently published report, this specific type of DFPP was described to be able to remove neurotransmitter receptor antibodies against ß-adrenergic and muscarinic receptors (linked to myalgic encephalomyelitis/chronic fatigue syndrome, ME/CFS) present in the blood of patients with post-COVID-19 syndrome [66]. The treatment alleviated symptoms of CFS in these patients.

The blood composition of people treated with DPFF using the INUSpheresis technology with a specific filter (TKM58) has recently been shown to change significantly [69]. A decrease was found in the concentration of albumin, *γ*-globulins, triglycerides, total cholesterol, HDL-cholesterol, LDL-cholesterol, liporotein(a), ferritin, fibrinogen, IgG, IgM, IgA, total protein, INR, quick, platelets and an increase in erythrocytes, haematocrit and leukocytes. Furthermore, proteomics showed significant changes in the concentration of apolipoprotein-related proteins, parameters of the coagulation system, immunoglobulins, parameters of the complement system and other inflammation-related proteins.

Human blood consists about 55% of plasma which is composed of 91% of water, 7% of proteins (i.e., 57% albumin, 38% globulins, 4% fibrinogen and 1% prothrombin) and 2% of other solutes [70]. The composition of the human body is also characterized by a high water content. An adult human consists of about 40 L (men) and 30 L (women), respectively, of water [71,72]. This total body water can be subdivided into intracellular water (ICW) and extracellular water (ECW). Men have about 25 L of ICW and 15 L of ECW, women have 17 L of ICW and 11 L of ECW, and the ECW-to-ICW ratio (which can be determined by bioelectrical impendence analysis) increases with age [72].

Water in the tissue and blood exists in the form of free and bound water. Around dissolved ions or dipoles, the dipoles of the water molecules are oriented and form a hydration shell. The hydration shell of ions contains strongly bound water in the first hydration layer, and less bound water in the further layers. In case of proteins, water plays an essential role for folding, stability, and binding with other molecules [73]. Single water monomers interact with each other, forming water clusters of the form (H_2_O)*_n_* which can be neutral, protonated, deprotonated or auto-ionized [74,75]. Water in the blood (plasma) and tissue is present in a variety of structures and states, which makes water so special in biological systems.

Since the physicochemical properties of water can change when passing through a filter or when the concentration of substances in the water change, we hypothesized that such changes in the water of the blood plasma would also have to occur with blood washing using DFPP. In order to verify this experimentally, we carried out corresponding measurements using near-infrared (NIR) spectroscopy before and after a specific DFPP treatment (INUSpheresis with the TKM58 filter) in one subject as a case study. It has not previously been investigated how DFPP affects the water in the tissue and what water properties the eluate has.

## 2. Material and Methods

### 2.1. Double-Filtration Plasmapheresis Treatments and Spectroscopic Measurements

DFPP INUSpheresis (with TKM58 filter) treatments were performed on a 39-year-old man (first author, FS) at a private clinic in Switzerland in February 2022 on two different days, one day apart. Each treatment lasted about 2.5 h. No official ethical approval was necessary to conduct the measurements and report the results since it is a case report (Kantonale Ethikkommission, Kanton Zürich) and measurements were conducted by and on the first author (FS). The DFPP treatment was performed as part of a routine medical treatment.

A portable and ultra-compact NIR spectrometer (MicroNIR 1700, Viavi, Milpitas, CA, USA) was used to measure the diffuse reflectance spectra of the hand palm of the left hand and of the eluate. Measurements were performed in the spectral range 950–1650 nm with a nominal spectral resolution of 6.25 nm, an integration time of 11.7 ms and an average of 1000 scans for each spectrum to ensure an optimal signal-to-noise ratio. 15 consecutive measurements were performed for each measurement. Measurements were conducted in the morning one day before the first DFPP treatment (day 1), in the morning on the day of the first DFPP treatment (day 2), 1 h after the first DFPP treatment (day 2), in the morning on the day of the second DFPP treatment (day 4), 1 h after the second treatment (day 4) and in the morning one day after the second DFPP treatment (day 5). For each measurement it was ensured that the hand had subjectively the same temperature and the NIR spectrometer had also always the same temperature (*T* = 31.5 °C). Furthermore, the eluate of the first and second DFPP treatment was also measured by placing the NIR spectrometer on the plastic bag with the eluate inside and also measuring the spectrum of the plastic bag as a reference. This measurement was performed at an instrument temperature of *T* = 35.4, 35.5 and 35.6 °C.

### 2.2. Data Analysis

First, the spectra of the hand tissue and eluate measurements were pre-processed applying the SNV (standard normal variate) algorithm for baseline correction. SNV normalization was performed on both datasets separately.

For the analysis of the hand tissue measurements, the difference spectra (**X**_Diff_) were calculated for the first and second DFPP treatment according to

(1) $$X_{\mathrm{Diff}}^{1\mathrm{st}\;\mathrm{DFPP}}\left(A_{1}\right)=X\left(\mathrm{after}\;1^{\mathrm{st}}\;\mathrm{DFPP}\;\left(\mathrm{day}\;2\right)\right)-X\left(\mathrm{before}\;1^{\mathrm{st}}\;\mathrm{DFPP}\;\left(\mathrm{day}\;2\right)\right)$$

(2) $$X_{\mathrm{Diff}}^{1\mathrm{st}\;\mathrm{DFPP}}\left(A_{2}\right)=X\left(\mathrm{after}\;1^{\mathrm{st}}\;\mathrm{DFPP}\;\left(\mathrm{day}\;2\right)\right)-X\left(\mathrm{before}\;1^{\mathrm{st}}\;\mathrm{DFPP}\;\left(\mathrm{day}\;1\right)\right)$$

(3) $$X_{\mathrm{Diff}}^{2\mathrm{nd}\;\mathrm{DFPP}}\left(B_{1}\right)=X\left(\mathrm{after}\;2^{\mathrm{nd}}\;\mathrm{DFPP}\;\left(\mathrm{day}\;4\right)\right)-X\left(\mathrm{before}\;2^{\mathrm{nd}}\;\mathrm{DFPP}\;\left(\mathrm{day}\;4\right)\right)$$

(4) $$X_{\mathrm{Diff}}^{2\mathrm{nd}\;\mathrm{DFPP}}\left(B_{2}\right)=X\left(\mathrm{after}\;2^{\mathrm{nd}}\;\mathrm{DFPP}\;\left(\mathrm{day}\;5\right)\right)-X\left(\mathrm{before}\;2^{\mathrm{nd}}\;\mathrm{DFPP}\;\left(\mathrm{day}\;4\right)\right)$$

while the indices A_1_ and A_2_ (and B_1_ and B_2_, respectively) refer to the calculation of the difference spectra for the 1^st^ DFPP (2^nd^ DFPP, respectively) based on the data measured on the same day (A_1_, B_1_) and 24 h apart (A_2_, B_2_). With this approach, the spectroscopic measurement after the DFPP treatments were compared to two baselines (i.e., at the same day and before/after 24 h).

For the analysis of the eluate measurements, the spectrum of the empty plastic bag containing the eluate was removed from the spectrum of the eluate measured through the plastic bag. With this, the spectra of the eluate itself was obtained.

To analyze the water spectral changes, the aquaphotomics approach was used [76,77,78,79,80,81]. 12 spectral regions of particular interest in the region of the 1st overtone of water (C*i*, *i* = 1–12; Water Matrix Absorbance Coordinates, WAMACS) were analyzed in particular by calculating the absorbance of the difference spectra in these 12 spectral regions and visualizing the results on aquagrams to determine the specific water absorbance spectral pattern [76,77]. A listing of the WAMACS with the corresponding water properties can be found in Table 1 in Muncan et al. [79].

Data processing and visualizations were carried out in Matlab (R2017a, MathWorks, Inc., Natick, MA, USA) and R (2022.02.0).

## 3. Results

**Figure 1:**
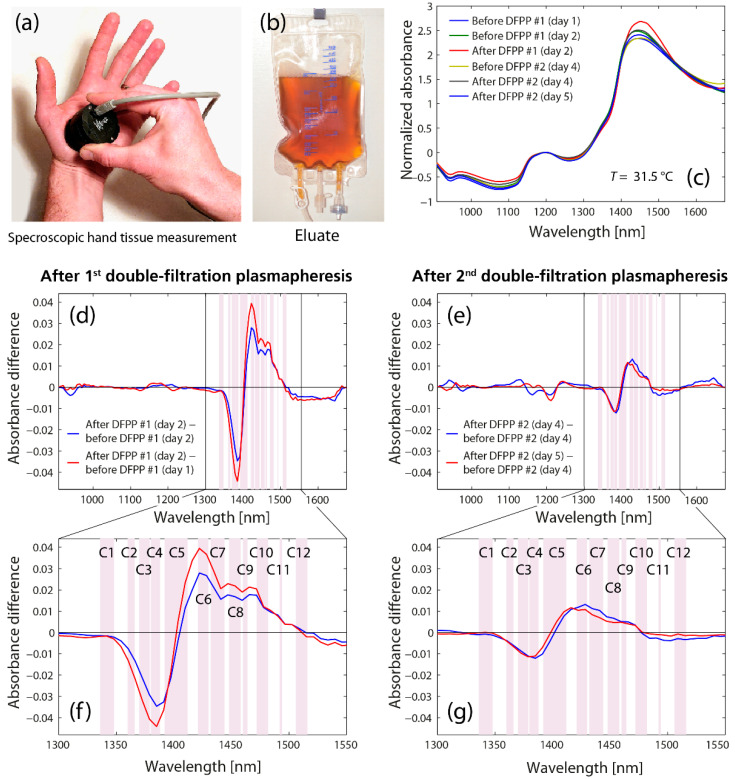
(**a**) Measurement performed on the palm of the left hand. (**b**) Bag with eluate extracted during the first DFPP treatment. The yellowish-brown color is striking, which is due to a high concentration of filtered substances. Normal blood plasma is clearer and more yellowish. (**c**) Raw spectra. (**d**) and (**e**) Difference spectra of the tissue measurements after the first ($X_{\mathrm{Diff}}^{1\mathrm{st}\;\mathrm{DFPP}}\left(A_{1}\right)$, $X_{\mathrm{Diff}}^{1\mathrm{st}\;\mathrm{DFPP}}\left(A_{2}\right)$) and second ($X_{\mathrm{Diff}}^{1\mathrm{st}\;\mathrm{DFPP}}\left(B_{1}\right)$, $X_{\mathrm{Diff}}^{1\mathrm{st}\;\mathrm{DFPP}}\left(B_{2}\right)$) DFPP treatment. (**f**,**g**) show zoomed-in regions with the 12 WAMACS. A listing of the WAMACS with the corresponding water properties can be found in Table 1 in Muncan et al. [79].

The spectroscopic analysis of the hand tissue before and after the DFPP treatments revealed clear differences in the spectral features in the first overtone spectral region of water, i.e., in the region of the 12 WAMACS (Figure 1). The corresponding aquagram (Figure 3a) shows the strongest increase in C6 (1421–1430 nm), corresponding to the water hydration band [79], the H–O–H bending mode, as well as the O-H stretch vibration mode, linked to the hydrogen bound strength of the water molecules [82]. The strongest decrease was found to be in C4 (1380–1388 nm), corresponding to water hydration shells (OH-(H_2_O),_1_,_4_), hydrated superoxide water clusters (O_2_-(H_2_O_4_)) and the H_2_O symmetrical stretch vibration (2*ν*_1_) [79]. In general, there was a decrease in C2 (1360–1366 nm) to C4 (1380–1388 nm) (linked to weaker H-bounded water) and an increase in C5 (1392–1412 nm) to C11 (1492–1494 nm) (linked to free water molecules and small water clusters). Interestingly, this specific change in spectral properties was evident after the first as well as the second DFPP treatment, whereas the first treatment caused the most pronounced effect. After the first DFPP treatment, the number of small and large water clusters increased, whereas after the second DFPP treatment, only the number of small water clusters increased while the larger ones decreased slightly.

**Figure 2:**
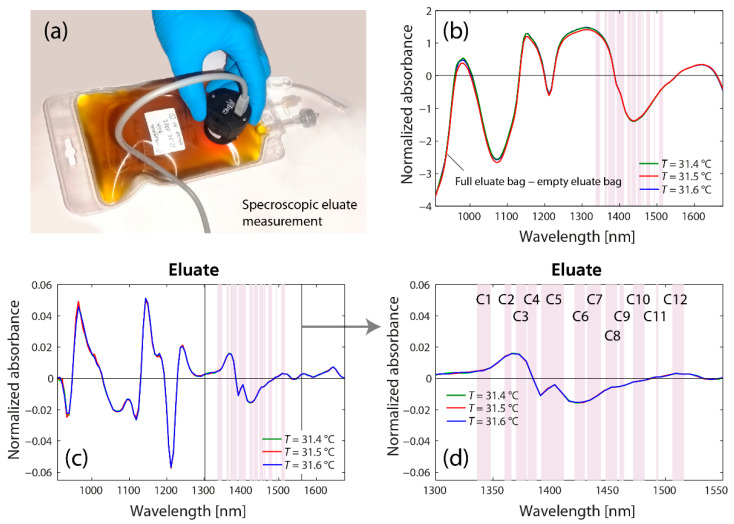
(**a**) Measurement of the eluate. (**b**) Raw spectra. (**c**) Difference spectra. (**d**) Zoomed-in part of (**c**) highlighting the region with the 12 WAMACS.

**Figure 3:**
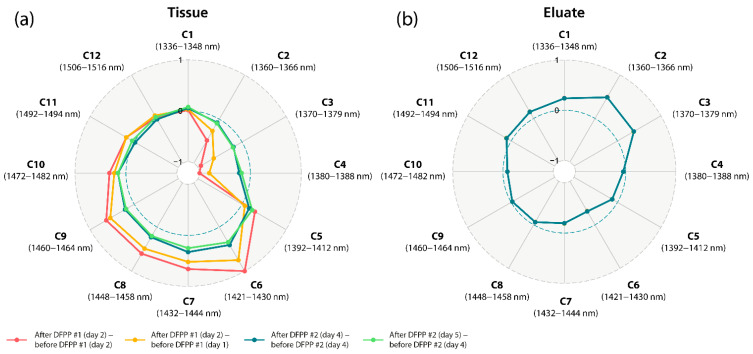
Aquagrams of the (**a**) tissue and (**b**) eluate spectra. The blue dashed circles refer to the zero baseline indicating no changes in the difference spectra. Note the complementary specific water absorbance spectral pattern of both aquagrams.

The spectroscopic analysis of the eluate obtained after the first and second DFPP treatment also revealed clear differences in the spectral features in the first overtone spectral region of water (Figure 2). The corresponding aquagram (Figure 3b) shows the strongest increase in absorbance at C2 (1360–1366 nm), corresponding to water salvation shells (OH-(H_2_O)_1,2,4_) and C3 (1370–1379 nm), corresponding to symmetrical and asymmetrical stretching vibration of water molecules (*ν*_1_  +  *ν*_3_) [79]. The strongest decrease in absorbance was at C6 (1421–1430) associated with the water hydration band, as well as the H-O-H bending mode and O-H stretch vibration mode. Fascinatingly, the specific water absorbance spectral pattern of the eluate is thus complementary to that measured in the tissue.

**Figure 4:**
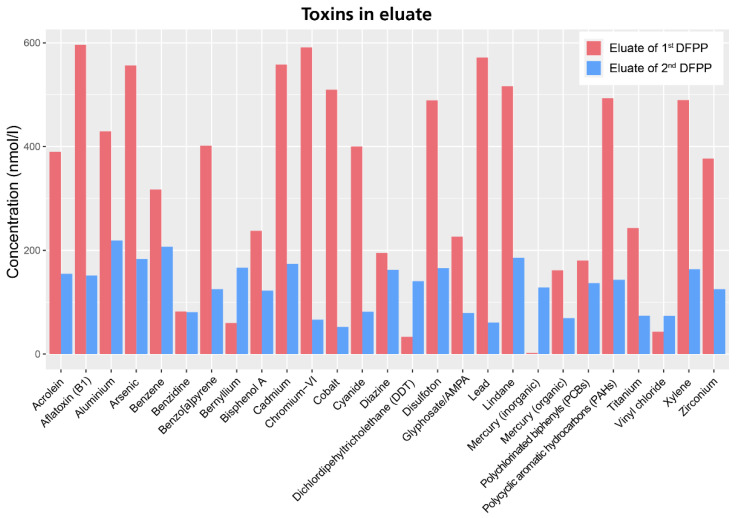
Concentration of toxins detected in the eluate of the first and second DFPP treatment. The first eluate showed a high amount of toxins whereas the amount was significantly reduced in the second eluate, highlighting the ability of DFPP to remove toxins from the blood/tissue.

To complement the aquaphotomics-based NIR spectral analysis, the eluate obtained from both DFPP treatment was also analyzed for toxic ingredients (IGL Labor GmbH, Wittbek, Germany). The analysis revealed the presence of several toxins (Figure 4). The ten highest detected concentrations were from aflatoxin B1 (596.2 nmol/L), chromium-VI (591.0 nmol/L), lead (571.6 nmol/L), cadmium (558.1 nmol/L), arsenic (556.1 nmol/L), lindane (516.3 nmol/L), cobalt (509.5 nmol/L), polycyclic-aromatic-hydrocarbons (493.1 nmol/L), disulfoton (489.2 nmol/L) and aluminum (429.3 nmol/L). The concentration of toxins was generally significantly lower in the eluate from the second DFPP treatment compared to the first one. The concentration of some toxins increased after the second DFPP treatment (DDT, mercury, vinyl chloride), indicating that the first DFPP treatment most probably caused a diffusion gradient from the tissue to the blood, releasing these toxins from the tissue into the blood. The detoxification with DFPP is a multi-stage process whereby different compartments in the human organism are cleaned.

## 4. Discussion and Conclusions

In this study, we have shown that the water properties in the tissue change after DFPP treatment (INUSpheresis with TKM58 filter). DFPP caused an increase in free water molecules, small water clusters and a decrease in hydroxylated water clusters, superoxides of water solvation shells and weaker H-bonded water. The opposite effect was observed in the eluates of both DFPP treatments.

In our opinion, these observations can be caused by two processes involved. First, the treatment filters out many molecules from the blood plasma to which water is bound in the form of hydration shells. This is the fraction of rather weakly H-bound water (which was reduced in the tissue after DFPP and was enriched in the eluates). Second, the process of filtration will cause water passing through the fine filter pores in a hydrophilic material to be structurally altered [83,84]. The formation of water clusters with stronger H-bonds is favored.

Our study is the first to date to investigate changes in tissue water after a DFPP treatment and also the first to perform a spectroscopic investigation of the eluates obtained by DFPP. In particular, it is also the first analysis of this kind concerning this specific type of DFPP, i.e., INUSpheresis with the TKM58 filter. We know of only one comparable study that investigated the dialysate after dialysis using NIR spectroscopy and the aquaphotomics approach [85]. This study showed that the absorption of 1398 nm and 1410 nm increased during dialysis. This finding is in line with our observation of an increase in this wavelength range after DFPP.

The changes in tissue water demonstrated by our case study suggest that the positive physiological effects of DFPP in general and of INUSpheresis with the TKM58 filter in particular, may be associated with improvements in water quality in blood and tissues related to the respective water molecular structures. Such an improvement in water quality could, for example, be associated later on with improved blood circulation and optimized metabolic processes. Our small study should serve as a stimulus to explore these possibilities through further, larger and more comprehensive studies.

It should be noted that our study is based on the measurements of a single person and two eluates. Generalizations of our results are only possible to a limited extent. As noted, our case study serves to stimulate further research on the interesting results and to show how an aquaphotomics-based analysis of NIRS data can be performed to investigate the effects of a DFPP.

## Data Availability

The data will be made available by the corresponding author, upon reasonable request.

## Sample Availability

Samples of the compounds are not available from the authors.
